# Harnessing controlled-environment systems for enhanced production of medicinal plants

**DOI:** 10.1093/jxb/erae248

**Published:** 2024-05-30

**Authors:** Ajwal Dsouza, Mike Dixon, Mukund Shukla, Thomas Graham

**Affiliations:** Controlled Environment Systems Research Facility, School of Environmental Sciences, University of Guelph, Guelph, ON, N1G 2W1, Canada; Controlled Environment Systems Research Facility, School of Environmental Sciences, University of Guelph, Guelph, ON, N1G 2W1, Canada; Department of Plant Agriculture, University of Guelph, Guelph, ON, N1G 2W1, Canada; Controlled Environment Systems Research Facility, School of Environmental Sciences, University of Guelph, Guelph, ON, N1G 2W1, Canada; University College Dublin, Ireland

**Keywords:** Circadian clock, controlled environment, functional genomics, LED light, medicinal plants, phytopharmaceuticals, plant factory, plant stress, specialized metabolites, vertical farming

## Abstract

Medicinal plants are valued for their contributions to human health. However, the growing demand for medicinal plants and the concerns regarding their quality and sustainability have prompted the reassessment of conventional production practices. Controlled-environment cropping systems, such as vertical farms, offer a transformative approach to production of medicinal plants. By enabling precise control over environmental factors, such as light, carbon dioxide, temperature, humidity, nutrients, and airflow, controlled environments can improve the consistency, concentration, and yield of bioactive phytochemicals in medicinal plants. This review explores the potential of controlled-environment systems for enhancing production of medicinal plants. First, we describe how controlled environments can overcome the limitations of conventional production in improving the quality of medicinal plants. Next, we propose strategies based on plant physiology to manipulate environmental conditions for enhancing the levels of bioactive compounds in plants. These strategies include improving photosynthetic carbon assimilation, light spectrum signalling, purposeful stress elicitation, and chronoculture. We describe the underlying mechanisms and practical applications of these strategies. Finally, we highlight the major knowledge gaps and challenges that limit the application of controlled environments, and discuss future research directions.

## Teaser

This review explores how controlled-environment systems (e.g. vertical farms) improve medicinal plant production. We propose environmental control strategies to increase bioactive phytochemicals in medicinal plants and highlight research gaps.

## Introduction

Medicinal plants (MPs) have improved human health and helped combat diseases throughout history (Raskin *et al.*, 2002; He *et al*., 2021; Lobato Gómez *et al.*, 2021). Today, MPs continue to support human health through various forms (Table 1), serving as the primary medical intervention for >80% of the global population. Moreover, ~65% of pharmaceutical drugs are derived or isolated from plants (Newman and Cragg, 2020). In recent years, the demand for MPs and their products has risen, owing largely to the growing interest in alternative medicine and the quest for novel therapeutics. As a result, the global market for herbal medicine has seen a rise in its commercial and pharmaceutical importance and one source suggests it could reach US$305 billion by 2029 (Market Data Forecast, 2024). The rich reservoir of bioactive phytochemicals in MPs holds untapped potential for promoting wellness and delivering novel clinical treatments (Howes *et al.*, 2020). However, the growing demand for MPs is exerting pressure on wild populations, raising sustainability concerns of these valuable botanical resources, and compromising the quality of MP products.

**Table 1.** Description and examples of medicinal plant products

Category	Description	Form	Example	Outlet
Pharmaceuticals	Chemical compounds derived from plants in a highly purified form	Isolated compounds	Paclitaxel, artemisinin, vinblastine, camptothecin	Rx
Botanical drugs	Clinically validated products for the treatment of diseases that contain ingredients from plants	Topicals, pills, solutions, powders	sinecatechins (Veregen^®^), crofelemer (Mytesi™), Polyphenon E^®^	Rx, OTC
Dietary supplements	Botanical preparations consumed by mouth for nutritional supplementation and health benefits	Tablets, capsules, soft gels, liquids, powders	Ashwagandha root, Echinacea extract, *Ginkgo biloba* extract, Rhodiola root powder	OTC
Recombinant products	Molecules produced using genetically modified plants	Proteins, vaccines, antibodies	CoVLP vaccine, ZMapp, Taliglucerase alfa (Elelyso^®^)	Rx, mostly experimental

Abbreviations: Rx, prescription drugs; OTC, over-the-counter products.

After Raskin *et al.* (2002), with permission from Elsevier.

**Fig. 1.**
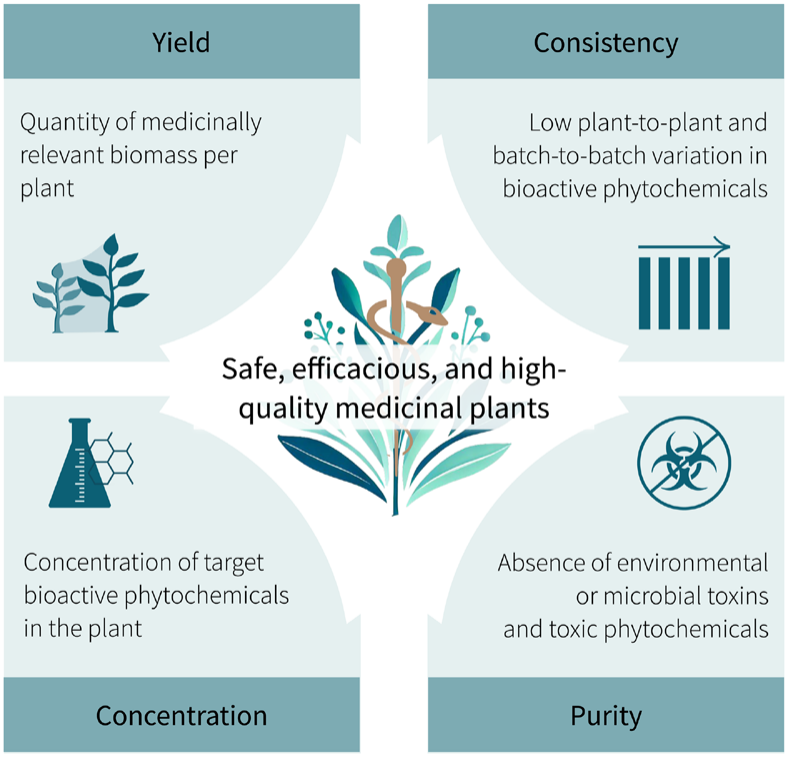
Critical quality attributes of safe, efficacious, and high-quality medicinal plants.

Conventional production practices, including field cultivation and wild harvesting, can compromise the critical quality attributes of MPs (Fig. 1). Medicinal plants grown in fields or natural habitats are subject to fluctuating environmental conditions, pests, and pathogens, resulting in inconsistent biochemical profiles (Bent and Ko, 2004; Ferrari, 2010; Anjali *et al.*, 2023). Moreover, MPs in fields and wild habitats are exposed to microbial toxins, pollutants, heavy metals, and herbicides/pesticides (Canter *et al.*, 2005; Jordan *et al.*, 2010). Unsustainable and exploitative harvesting threatens ~10 000 valuable MP species to extinction (Aslam *et al.*, 2017). These threats to plant populations, quality, and sustainability caused by conventional production or harvesting practices pose major challenges for the development of novel botanical drugs (Atanasov *et al.*, 2015). Given these challenges and the anticipated increase in demand for MPs, it is imperative to redefine production technologies to produce safe, clean, reliable, and consistent MPs and related products.

**Fig. 2.**
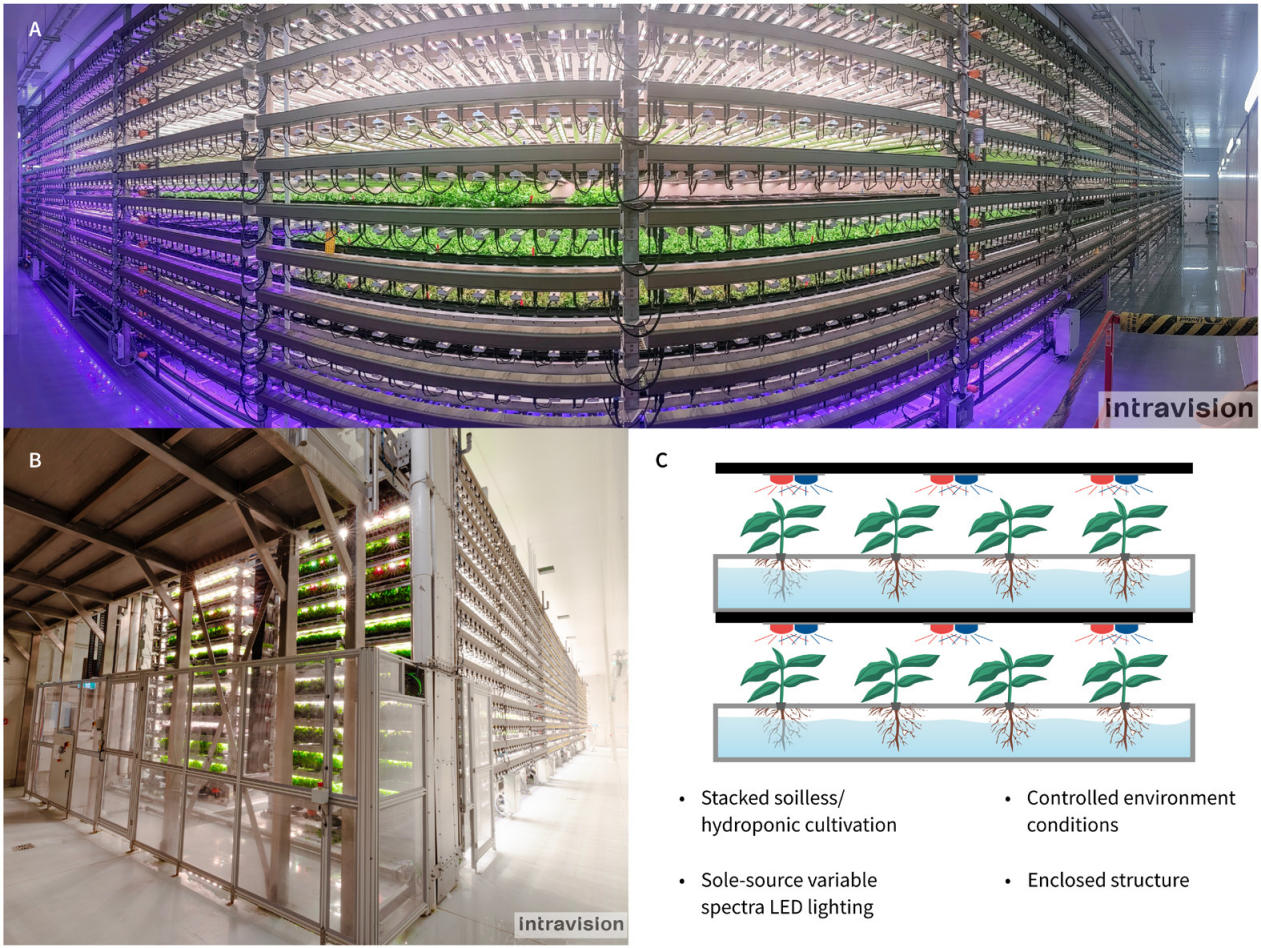
Controlled-environment plant production systems, also called vertical farms or plant factories. Images (A) and (B) courtesy of the Intravision Group, with permission.

Controlled-environment systems are a transformative approach in modern crop production, offering a more consistent or standardized cultivation of MPs. These systems, often referred to as vertical farms or plant factories, involve indoor, stacked, soil-less plant cultivation under LED lights (Fig. 2) (van Delden *et al.*, 2021). Controlled-environment systems exercise precise control over environmental factors relevant to plant growth, such as light intensity and spectrum, temperature, CO_2_ concentration ([CO_2_]), humidity, water, nutrients, and airflow. By optimizing these factors, controlled-environment systems enable year-round cultivation, improve plant quality, eliminate the need for chemical pesticides, and maximize water and nutrient use efficiency. These strengths underscore the significance of controlled-environment systems in addressing the challenges of conventional practices and improving MP production.

The production of MPs in controlled-environment systems, a practice we refer to as controlled-environment ‘phyto-pharmaculture’ (CEP), holds significant promise in tackling the key challenge in conventional MP production: environmental variability. By maintaining precise control over environmental conditions, CEP can facilitate the seed-to-pill standardization of MPs, a crucial requirement in the pharmaceutical industry (Schmidt *et al.*, 2007). Moreover, optimizing the environment can enhance plant biomass and yield in MPs (Zobayed *et al.*, 2005; Fonseca *et al.*, 2006; Stutte, 2016).

Tailoring the environment to optimize bioactive compound production in specific MP species is challenging due to complex environmental and phytochemical interactions. To effectively implement CEP, we require strategies that leverage our knowledge of plant physiology and plant–environment interactions to manipulate growing conditions for enhancing bioactive phytochemical levels in MPs. This underscores the need for targeted research to optimize controlled environments for high-quality MP production.

This review brings together the knowledge on the contribution, prospective strategies, and outlooks of CEP to produce high-quality, safe, and standardized MPs, with enhanced levels of bioactive phytochemicals. First, we demonstrate how CEP can overcome the challenges of conventional production in delivering the critical quality attributes of MPs. Next, based on fundamental plant biology, we propose environmental control strategies to enhance the levels of target bioactive compounds, which include increasing photosynthetic carbon assimilation, light spectrum signalling, stress elicitation, and circadian regulation of environmental conditions. We also delve into the underlying mechanisms, practical applications, and potential synergistic effects of the proposed strategies. Lastly, we highlight challenges impeding the application of CEP and discuss future research directions.

## Controlled-environment phyto-pharmaculture improves medicinal plant production compared with conventional production

### Consistency

Diurnal and seasonal environmental fluctuations in both wild harvesting and field cultivation cause significant variations in the bioactive compounds of MPs (Southwell and Bourke, 2001). For instance, the concentration of hypericin and pseudohypericin in *Hypericum perforatum* increased 40-fold, from <100 ppm in the winter to >4000 ppm in the summer (Southwell and Bourke, 2001). Similarly, the concentration of cardenolides in *Digitalis obscura* leaves (used medicinally as cardiac glycosides, compounds that generally act to alter heart contractions) is lower in the spring and autumn, and peaks in the summer (Roca-Pérez *et al.*, 2004). Geographical location further exacerbates variations in phytochemicals, as observed in Ashwagandha (*Withania somnifera*) (Kumar *et al.*, 2007), *Artemisia annua* (Wallaart *et al.*, 2000), and *Salvia miltiorrhiza* (Zhao *et al.*, 2016; Zhang *et al*., 2019). A variable environment leads to inconsistencies in the active ingredients of commercial MP products, compromising their purity, potency, and safety (Awang *et al.*, 1991; Cui *et al.*, 1994; Harkey *et al.*, 2001). This issue is exemplified by an analysis of 25 commercial ginseng products, in which measured marker compounds ranged from 10.8% to 327.7% of the labelled concentrations (Harkey *et al.*, 2001).

A standardized metabolite profile is essential for the efficacy and safety of herbal medicine (Ernst, 1998; Fong, 2002). Inconsistent levels of bioactive compounds in botanical drugs and dietary supplements can hinder reproducibility and lead to unpredictable therapeutic effects, health hazards, and adverse interactions with other medications (Angell and Kassirer, 1998; Shipkowski *et al.*, 2018). Therefore, strict environmental control during cultivation is necessary to ensure consistent bioactive phytochemical profiles in MPs.

Consistency is particularly critical for plant-based medicines containing multiple bioactive compounds from one or more plant species. These multiple compounds work synergistically across various systems and pathways to deliver the therapeutic benefits (Liu, 2003; Rasoanaivo *et al.*, 2011; Solowey *et al.*, 2014). A prime example is PHY906—a pharmaceutical-grade formulation of four herbs: *Scutellaria baicalensis*, *Glycyrrhiza uralensis*, *Paeonia lactiflora*, and *Ziziphus jujuba*—known as Huang-Qin-Tang in Traditional Chinese Medicine. In addition to its extensive use in traditional medicine as a general remedy, clinical trials have demonstrated the efficacy of PHY906 as an adjuvant for cancer chemotherapy (Lam *et al.*, 2010; Liu and Cheng, 2012) and as an enhancer of the anticancer activity of the chemotherapy drug CPT-11 (Liu *et al.*, 2004). PHY906 contains >60 bioactive compounds (Ye *et al.*, 2007) that work through multiple mechanisms across various signalling pathways to provide PHY906’s adjuvant effects (Lam *et al.*, 2010). Inconsistent metabolite contents of the constituent plants due to varying growth environments present challenges in testing and delivering PHY906, and similar other plant formulations with multiple bioactive compounds (Callaway, 2010).

CEP can significantly enhance phytochemical consistency through precise control of environmental factors such as temperature, CO_2_, humidity, light, and nutrient levels. By eliminating fluctuating weather, variable soil quality, and pests, CEP ensures consistent phytochemical composition in MPs. Furthermore, species-specific environment ‘recipes’ can be consistently applied in CEP systems, regardless of geographic region or local climate. Therefore, CEP provides a superior method for cultivating MPs with consistent phytochemical profiles, ensuring standardized pharmacological effects, improving safety, and facilitating reliable clinical trials.

### Concentration and yield

Compared with CEP, wild harvesting and field cultivation limit plant biomass yield due to the unpredictable environment and exposure to pests and diseases (Chakraborty and Newton, 2011; Dixon, 2012). Moreover, scalability is constrained in wild harvesting due to extinction threats, while field cultivation demands scarce arable land.

Optimizing the environment in CEP is a promising approach for augmenting biomass and phytochemical yield in MPs. For instance, the biomass yield and concentrations of targeted therapeutic metabolites in St. John’s Wort (*Hypericum perforatum* L.) grown under an optimized controlled environment were 5–10 times higher than in field-grown plants (Mosaleeyanon *et al.*, 2005, 2006). Similarly, Echinacea (*Echinacea purpurea*) grown in a controlled environment showed increased levels of cynarin, caftaric acid, echinacoside, and chicoric acid (Zheng *et al.*, 2006). Elevated [CO_2_] (700 ppm) increased the yield of anti-cancer and anti-viral phytochemicals in *Hymenocallis littoralis* by 75% (Idso *et al.*, 2000). These findings highlight CEP’s potential to boost medicinal component yields through environmental optimization.

Optimal temperature, high light intensity, and extended photoperiods in CEP can accelerate the phenological development and shorten the growth cycle of plants (Stutte *et al.*, 2007; Poorter *et al.*, 2016; Watson *et al.*, 2018). For example, Goldenseal (*Hydrastis canadensis*) grown in a controlled environment under elevated [CO_2_] yielded double the fresh biomass in 15 weeks, compared with the 2–3 years required for field-grown plants (Adelberg *et al.*, 2016). Similarly, Chinese liquorice (*Glycyrrhiza uralensis*) cultivated under an optimized light spectrum and elevated [CO_2_] for 3–6 months can yield glycyrrhizin concentrations comparable with plants grown in open fields for 3–4 years (Afreen *et al.*, 2005). Efficient use of cultivation volume in CEP by stacking plant production also increases crop productivity. Therefore, through environmental optimization, efficient resource use, and accelerated growth cycles, CEP can significantly boost biomass yield, phytochemical concentration, and productivity of MPs.

### Purity

Safety is paramount in medicinal products. Yet, conventional production poses safety concerns for MPs due to the risk of contamination. Field-grown MPs are exposed to chemical fertilizers, pesticides, microbial contaminants, and heavy metals, leading to toxic residues in end-products (Zuin and Vilegas, 2000; Bent and Ko, 2004; Xue *et al.*, 2008a, b). Wild-harvested MPs are also susceptible to contamination and misidentification, which can lead to adverse effects upon consumption (Woo *et al.*, 2012). As a result, conventionally produced MPs fail to meet the stringent safety standards required for medicinal products, jeopardizing consumer health (Beigel *et al.*, 1998; Slifman *et al.*, 1998).

In contrast, CEP grows plants indoors under sanitary conditions, adhering to strict biosecurity protocols that prevent pests and diseases, eliminating the need for chemical control agents. Furthermore, hydroponic cultivation allows precise control over nutrient delivery, reducing excess fertilizer residues on the plants. Monitoring the quality of growing substrate, water, and fertilizers can prevent heavy metal contamination. CEP allows the assessment of critical contamination pathways during production, to ensure the purity and safety of MPs.

## Environmental control strategies to improve medicinal phytochemical yield

**Fig. 3.**
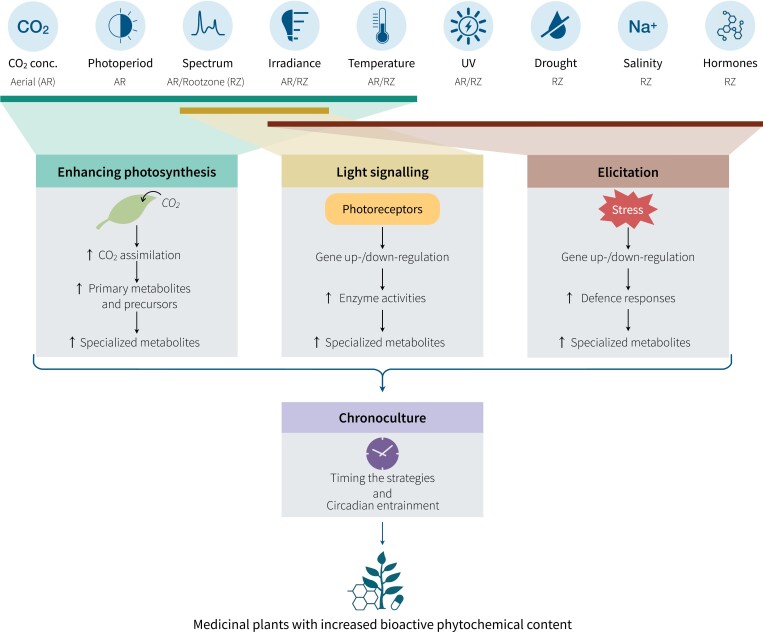
Strategies for enhancing bioactive phytochemical contents in medicinal plants through environmental control and manipulation of the aerial (AR) and root-zone (RZ) environment in controlled-environment phyto-pharmaculture. A suite of environment factors can be optimized to enhance plant photosynthetic carbon assimilation, thereby stimulating biosynthesis of specialized metabolites (SMs). Modulating light spectra and intensity can harness photoreceptor-driven light signalling mechanisms to enhance SMs. Through elicitation, environmental stress can also be applied to induce defence responses triggering SMs. These three strategies can be timed based on circadian biology to maximize plant responsiveness.

Controlled manipulation of environmental factors unlocks untapped potential to stimulate the production of target bioactive phytochemicals in plants. However, to achieve this predictably and consistently, we need strategies leveraging a mechanistic understanding of plant responses to specific environmental cues. Drawing from research in photobiology, photosynthetic carbon assimilation, plant defence, and circadian biology, we propose environmental control strategies in CEP that leverage plant biology to enhance the production of medically valuable specialized metabolites (illustrated in Fig. 3).

### Harnessing photobiology

Light is pivotal for plant growth, signalling, and metabolism. By manipulating key light parameters—spectrum, intensity, and photoperiod—growers can modulate plant development and biochemical composition. This is achieved through light-dependent modes of action such as an increase of photosynthetic carbon assimilation, photoreceptor-mediated regulation of biochemical pathways, circadian regulation, stress elicitation, and photomorphogenesis.

The light spectrum significantly influences the biosynthesis of specialized metabolites (SMs) in plants by activating specific photoreceptors—cryptochromes and phototropins by blue and UV-A light, UVR8 by UV-B light, and phytochromes by red and far-red light. These photoreceptors regulate light-induced metabolic responses by controlling gene expression and enzyme activity associated with specialized metabolism via transcription factors such as HY5 or PIFs (Tuan *et al.*, 2013; Thwe *et al.*, 2014; Liu *et al.*, 2018; Contreras-Avilés *et al.*, 2024). A mechanistic knowledge of how light spectra affect various SM biosynthetic pathways can be applied practically in CEP as spectral treatments.

Within photosynthetically active light (400–700 nm), red and blue light significantly regulate SM pathways. In controlled-environment systems, spectral treatments can be applied using variable-spectra LED lights to stimulate SM production in MPs. For instance, red and blue light increased the artemisinin content in *A. annua* over white light (Zhang *et al*., 2018). Similarly, blue light increased vinblastine and vincristine by 9-fold in *Catharanthus roseus* compared with white light (Nagy *et al.*, 2023). Blue light also enhanced the concentration of bioactive phytochemicals in *H. perforatum* flowers compared with red or white light (Karimi *et al.*, 2022). Overall, blue light consistently enhances photoprotective SM without impairing plant growth and development (Landi *et al.*, 2020). Conversely, red light alters plant morphology and physiology with varied effects on specialized metabolism (Landi *et al.*, 2020).

Wavelengths beyond the traditionally defined photosynthetically active range (400–700 nm), specifically far-red (700–800 nm) and UV-A/UV-B (280–400 nm), interact with red and blue wavelengths to activate plant metabolic pathways via photoreceptor-mediated mechanisms (Ballaré, 2014). For instance, the red to far-red ratio is a key signal enhancing the production of flavonoids and phenolics in plants through phytochrome signalling (Tegelberg *et al.*, 2004; Qaderi *et al.*, 2015; Fu *et al.*, 2016; Bae *et al.*, 2017). UV-A light modulates the plant responses through different photoreceptors based on the wavelength range. Long-wave UV-A (350–400 nm) acts on cryptochromes to induce flavonoid production, while short-wave UV-A (315–350 nm) induces SM responses through UVR8 photoreceptors (Rai *et al.*, 2020; Zhang *et al*., 2024). UV-B light, however, typically elicits a stress response in plants, up-regulating the biosynthesis of protective SMs, such as phenolics, flavonoids, alkaloids, terpenoids, and glucosinolates (Yadav *et al.*, 2020; Meyer *et al.*, 2021; Contreras-Avilés *et al.*, 2024).

Applying tailored spectra, comprising specific combinations of wavelengths, could precisely modulate plant metabolism, thereby enhancing the production of targeted bioactive metabolites (Holopainen *et al.*, 2018). However, the influence of light spectra on the SMs varies with plant species, phenological stage, light intensity, and interactions with other variables (Taulavuori *et al.*, 2016, 2018). Therefore, the successful application of light spectra for enhanced or controlled SM production requires further research to unravel species-specific responses.

High light intensity and extended photoperiod can trigger a stress response inducing the production of protective SMs. For instance, high light intensities exceeding 900 μ mol m^–2^ s^–1^ increased flavonoid content in *Orthosiphon stimaneus* (Ibrahim and Jaafar, 2012) and *Passiflora suberosa* (Ni *et al.*, 2020). Similarly, under elevated [CO_2_], the concentration of SMs was maximized at high light intensities for *Populus tremuloides*, *Betula papyrifera*, and *Acer saccharum* (McDonald *et al.*, 1999). A longer photoperiod is typically correlated with increased SMs in plants (Carvalho *et al.*, 2010; Hoffman *et al.*, 2010), as seen with the anti-tumour agent camptothecin in *Ophiorrhiza pumila* (Lee *et al.*, 2020). This photoperiodic effect is possibly linked to the regulation of genes controlling photosynthesis, primary metabolism, and specialized metabolism (Hoffman *et al.*, 2010). Alternatively, it could be due to enhanced photosynthesis and photoprotection triggered under high daily light integrals.

LED lights in CEP offer a significant opportunity to enhance bioactive SMs by tailoring light recipes based on the effects of specific light treatments on metabolite pathways. However, our understanding of the synergistic and pleiotropic effects of manipulating multiple light parameters on medicinally relevant plant SM is limited. Light manipulation poses challenges due to its varying effects on SM production, depending on the plant species and their light tolerances. For instance, flavonoid content in *Epimedium pseudowushanense* and *Anoectochilus formosanus* peaked at low light intensities of 60–90 μmol m^–2^ s^–1^, declining thereafter (Ma *et al.*, 2010; Pan and Guo, 2016; Pan *et al.*, 2017). In contrast, flavonoid content peaked only at a high intensity of 900 μmol m^–2^ s^–1^ in *Orthosiphon stimaneus* (Ibrahim and Jaafar, 2012) and ~1350 μmol m^–2^ s^–1^ in *Passiflora suberosa* (Ni *et al.*, 2020). Similarly, under elevated [CO_2_], the concentration of bioactive compounds was maximized at high light intensities for *P. tremuloides*, *B. papyrifera*, and *A. saccharum* (McDonald *et al.*, 1999), while *Labisia pumila* showed maximum bioactive compounds only under low light (Ibrahim *et al.*, 2014). The changes in SM levels due to light intensity can also vary with the specific classes of SMs (Xu *et al.*, 2020). These challenges underscore the need for further research to understand how light affects SM pathways for reliable translation into CEP.

### Optimizing photosynthetic carbon assimilation

Plant photosynthesis leads to the production of primary metabolites, a substantial portion of which are channelled into specialized metabolic pathways. The metabolic pathways, such as shikimate, phenylpropanoid, mevalonate, malonate, and methylerythritol phosphate pathways, depend on precursors from primary metabolism from photosynthetically fixed carbon. For instance, the shikimate pathway, leading to the biosynthesis of aromatic amino acids and subsequently indolic and phenolic SMs, is estimated to consume >30% of the photosynthetically fixed carbon (Maeda and Dudareva, 2012). As such, a recent study showed that carbon newly fixed during photosynthesis can be routed towards phenylpropanoid metabolism, through *de novo* synthesis of the precursor phenylalanine via the shikimate pathway (Abadie *et al.*, 2024).

Increasing the rate of net photosynthetic carbon assimilation (NPCA) in plants can increase the activity of the Calvin cycle to augment the pool of photoassimilates, such as glyceraldehyde-3-phosphate, a key precursor for terpenoid synthesis. Moreover, elevated NPCA can increase carbon stores, thereby expanding the precursor pool for SM biosynthesis (Peñuelas and Estiarte, 1998; Li *et al*., 2017). A higher photosynthetic rate also increases the respiration rate, leading to higher NADPH and ATP levels needed for SM biosynthesis (Gonzalez-Meler *et al.*, 2004).

**Fig. 4.**
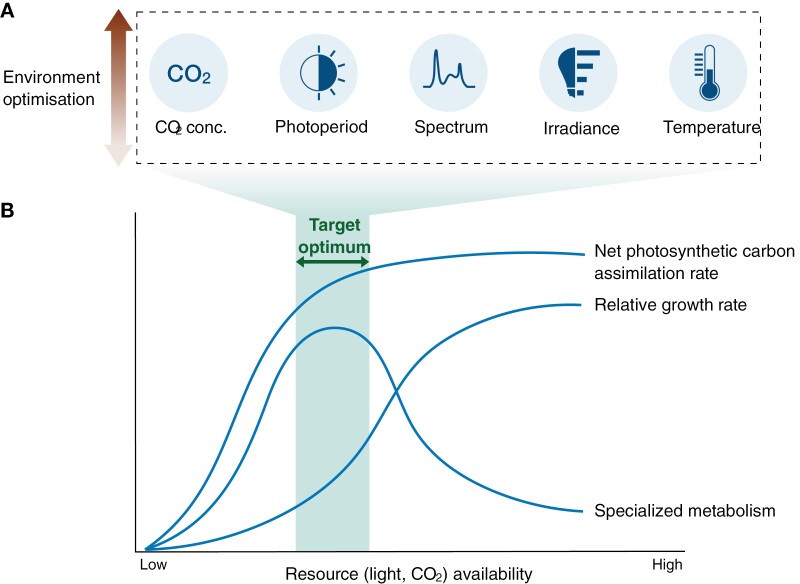
Enhancing net photosynthetic carbon assimilation (NPCA) to improve specialized metabolism by balancing growth rates. (A) Environmental factors that can be optimized in combination to increase NPCA. (B) The relationship between NPCA, growth, and specialized metabolism is non-linear, with specialized metabolism surging initially but declining beyond a saturation point, highlighting the target optimization zone.

In CEP, factors such as light spectrum, light intensity, photoperiod, [CO_2_], and temperature can be optimized individually or collectively to increase the NPCA in MPs, thereby boosting SM production (Fig. 4A). Elevated [CO_2_] has been shown to increase the rate of specialized metabolism and certain SM concentrations via enhanced photosynthesis and respiration (Matros *et al.*, 2006; Levine *et al.*, 2008; Stutte *et al.*, 2008; Ghasemzadeh and Jaafar, 2011; Li *et al*., 2017). For instance, the content of hypericin and pseudohypericin in St. John’s Wort was strongly correlated with the net photosynthesis rate under increased light intensity and [CO_2_] (Mosaleeyanon *et al.*, 2005). Besides SM, enhancing NPCA through elevated [CO_2_] and increased light intensity can also boost bioactive polysaccharides by augmenting the production of the glycolytic intermediate 3-phosphoglyceric acid (Raines, 2011; Pan *et al.*, 2020), a precursor for sugar and polysaccharide synthesis.

The hypothesis underpinning the strategy of augmenting SMs through increased NPCA rests on the premise that high NPCA expands the free carbon pool available for SM production. However, this idea is occasionally contradicted in the ecological literature (Chang *et al.*, 2016; Jia *et al.*, 2016). Increased resource (light and CO_2_) availability can accelerate the growth rates, utilizing much of the photosynthetically assimilated carbon, leaving little for specialized metabolism. The Growth–Differentiation Balance model extended to specialized metabolism (GDB_e_) suggests that specialized metabolism may initially increase with increasing carbon assimilation and growth (Fig. 4B) (Herms and Mattson, 1992; Mattson *et al.*, 2005). However, beyond the saturation point of carbon assimilation, the rate of specialized metabolism declines as the resources are preferentially allocated for growth (Neilson *et al.*, 2013; He *et al*., 2022). It is at lower growth rates that the carbon-derived intermediates could be effectively shunted towards specialized metabolism (Herms and Mattson, 1992). In controlled-environment production of food crops, NPCA is optimized to maximize growth rate and biomass yield. In contrast, the goal with MPs is to optimize the environment to maximize the rate of specialized metabolism, albeit at a growth rate possibly lower than the potential maximum (Fig. 4B).

A challenge with enhancing photosynthesis through environmental optimization is photosynthetic acclimation. Prolonged exposure to elevated [CO_2_] can down-regulate photosynthesis (Ainsworth and Long, 2005). Similarly, high light intensities can cause photoinhibition, reducing photosynthetic rates (Powles, 1984). Furthermore, at high light intensities or elevated [CO_2_], water or nutrients may become limiting if not properly managed, potentially decreasing photosynthesis. Much remains to be elucidated about reliably enhancing specialized metabolism by elevating NPCA without compromising plant growth. Investigating species- and organ-specific allocation of resources towards SMs under enhanced NPCA can provide insights into optimizing environments to augment SMs in MPs.

### Controlled elicitation

The production of SMs in plants is primarily a defence response to stressors. This defence response can be triggered in cultivated plants by intentionally simulated stress, a process called elicitation. The stress-inducing agents, or elicitors, include physical, chemical, or biotic stimuli, and can effectively augment the concentration of medicinally relevant SMs in plants (Thakur *et al.*, 2019). Water stress, for instance, increased hyperforin content in St. John’s Wort (Zobayed *et al.*, 2007), while low-dose UV-B radiation increased stigmasterol and sarsasapogenin in safed musli (*Chlorophytum borivillianum*) (Jaiswal *et al.*, 2023). High light intensity increased bioactive flavonoids and polysaccharides in *Dendrobium officinale* (Li *et al*., 2023). Elicitation is a highly effective and reproducible strategy for augmenting bioactive phytochemicals in MPs (Raskin *et al.*, 2002). The variety of effective elicitors highlights the potential for customizing elicitation strategies to specific plant species and SMs. In CEP, environmental elicitors such as high temperature, UV-B radiation, high-intensity light, and drought can be applied consistently and repeatably. Temperature and light stress can be imposed using environmental control systems and LED lighting, respectively, while UV-B stress can be imposed using specialized UV luminaires.

CEP allows for controlled, scheduled, and intermittent elicitation, which can enhance SM production under stress by providing stress-free recovery periods, as opposed to continuous stress. For instance, UV-B stress interspersed with recovery periods devoid of UV-B increased the accumulation of flavanols in Arabidopsis without impairing growth, compared with continuous UV-B exposure (Höll *et al.*, 2019). This response suggests the potential of intermittent elicitation with recovery periods in enhancing the effectiveness of elicitation in CEP. However, more research is needed on intermittent elicitation, including exploring intermittent elicitation with other stressors, such as high light and drought.

**Fig. 5.**
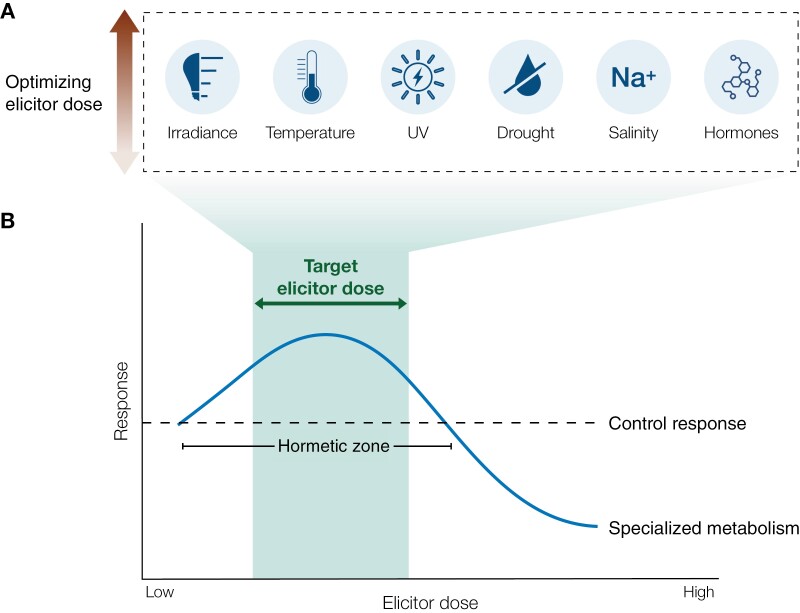
Optimizing elicitor dose based on the hormetic dose–response model to improve specialized metabolism as a response to elicitation. (A) Elicitors that can be optimized for elicitation in CEP. (B) Schematic of the hormetic dose–response relationship between elicitor dose and specialized metabolism, which suggests the presence of an ideal target elicitor dose within the hormetic zone of plant responses.

Recent research suggests a hormetic response in plant defence and SM production, where low to moderate stressor levels are beneficial, but high levels are harmful (Fig. 5B) (Hadacek *et al.*, 2011; Urban *et al.*, 2016; Vargas-Hernandez *et al.*, 2017). The hormetic dose–response model leads us to posit an optimal stressor dose that stimulates SM production without markedly impeding growth (Fig. 5). By determining and applying the ideal dose of elicitors, we can leverage the innate defence responses of plants for controlled and reproducible stimulation of bioactive SMs (Agathokleous *et al.*, 2019; Godínez-Mendoza *et al.*, 2023). As research continues to explore and optimize elicitation strategies, CEP systems are well positioned to implement these findings to enhance the value of MPs.

### Chronoculture—circadian regulation of medicinal plants

The circadian clock in plants regulates biological processes, ‘entraining’ physiological responses to environmental cues such as light, temperature, and humidity, and ‘gating’ these responses to specific times of day (Hotta *et al.*, 2007). For instance, UV-B light entrains the circadian clock by activating the clock genes through the photoreceptor UVR8 and photomorphogenic regulator COP1 (Fehér *et al.*, 2011; Horak and Farré, 2015). The circadian clock also gates the plant responses to UV-B, rendering plants less susceptible to brief UV-B stress during the day than at night (Takeuchi *et al.*, 2014). This circadian regulation of plant processes can be harnessed to improve crop production through ‘chronoculture’, which involves aligning horticultural management and cultivation practices with the plant circadian rhythms by applying treatments during optimal times of the day (Steed *et al.*, 2021).

Chronoculture can enhance the growth and medicinal attributes of MPs. Plant responses to abiotic elicitors such as temperature and drought vary throughout the day (Horak and Farré, 2015; Grinevich *et al.*, 2019; Liebelt *et al.*, 2019), such that SM production can be optimized by precisely timing elicitation. In addition to gating elicitor responses, circadian clocks also regulate plant metabolism and the metabolome in response to the diel environmental rhythms (Lu *et al.*, 2002; Kerwin *et al.*, 2011; Wang *et al.*, 2021). Diel environmental variation often acts as a cue for inducing time-of-day-dependent synthesis of SMs in plants (Liebelt *et al.*, 2019). For instance, end-of-day far-red light modulates the biochemical composition of plants, such as increasing phenols and decreasing alkaloids in tobacco (Tso *et al.*, 1970). Similarly, the effect of supplemental blue light on phenolic acids, flavonoids, and pigments in *Lactuca sativa* varies depending on the time of application within the diel cycles (Ouzounis *et al.*, 2015). Therefore, identifying and harnessing such time-of-day-dependent responses to diel environmental cues is another key chronoculture strategy for improving MPs. Studying MPs’ diurnal response patterns to specific elicitors is key to maximizing phytochemical production.

In CEP, environmental variables such as temperature, light, CO_2_, water availability, and humidity can be controlled in accordance with the period, amplitude, and phase of the plant’s circadian cycle to optimize growth and phytochemical content. Elicitors applied when plants are most responsive within the diel cycle can enhance metabolite content while minimizing growth impact (Hotta, 2021). Dynamic lighting, characterized by diel variation in light spectra at the start or end of the day, can also stimulate SM production (SharathKumar *et al.*, 2020). Changes in day length, detected by the circadian clock, closely govern plant responses to altered photoperiods. Aligning the exogenous light–dark cycles with the plant clock period can also improve plant NPCA (Dodd *et al.*, 2005).

Essentially, implementing chronoculture in CE systems involves timing treatments and synchronizing light and temperature with plant circadian rhythms. Put together, chronoculture, by applying environmental cues at optimal times, can fine-tune plant responses to increase biomass yield and SM content. However, understanding of the circadian regulation of SM, especially for commercial production of MPs in CEP, is limited. More research is needed to understand the mechanisms of circadian regulation of SM production and to tailor chronoculture protocols to different MP species and environmental factors.

### Root-zone manipulation

Controlled-environment systems predominantly use hydroponics [refer to van Delden *et al.* (2021) for a summary of various hydroponic systems] to grow plants, as it offers tight control of the plant root-zone (RZ) environment. Optimizing RZ factors, such as temperature, dissolved oxygen, nutrients, and salinity, can enhance the growth and SMs of MPs (Dayani and Sabzalian, 2017). Moreover, RZ treatments could outperform aerial treatments in stimulating SMs in MPs where the roots are the primary medicinal part.

Tailoring the nutrient composition of the hydroponic solution is a key RZ variable for steering SM biosynthesis and plant growth. Modifying the concentration and ratio of nitrogen, phosphorus, potassium, and micronutrients can affect enzyme levels, enzyme activity, and precursor availability for SM biosynthesis (Abdalla *et al.*, 2021; Corrado *et al.*, 2021a). Such tailored nutrient management has been effective in improving levels of SMs in *Mentha* sp. (Zeljković *et al.*, 2022) and *Echinacea* sp. (Zheng *et al.*, 2006; Ahmadi *et al.*, 2021).

In addition to nutrient manipulation, the proposed environmental control strategies can also be applied to RZs to elicit distinct shifts in SMs (Fig. 3). RZ temperature influences plant NPCA (He *et al*., 2009, 2013), and may improve SM biosynthesis, as discussed above. In contrast, suboptimal RZ temperatures can impede nutrient uptake and photosynthesis. Low and high RZ temperatures also induce stress, stimulating the production of phenolic compounds in *Coriandrum sativum* (Nguyen *et al.*, 2020). A low RZ temperature of 12 °C increased alkaloid production in *C. roseus* and *N. tabacum* with associated up-regulation of alkaloid pathway genes (Malik *et al.*, 2013). A low RZ temperature (15 °C) also up-regulated genes controlling the phenylpropanoid pathway, coupled with an increase in net photosynthesis in *N. benthamiana* (Son *et al*., 2023). However, with only limited studies, the mechanisms leading to increased SM production under low RZ temperatures are yet to be elucidated.

LEDs can be used for root illumination in soil-less hydroponic systems, activating signalling pathways that stimulate SM production (Hemm *et al.*, 2004). Root illumination with white, blue, and red wavelengths enhanced the accumulation of artemisinin in *A. annua* shoots and coumaroylquinic acid in *H. perforatum* leaves (Paponov *et al.*, 2023). These responses suggest root illumination as a potential CEP strategy for enhancing bioactive compound production.

RZ elicitors, such as salinity and pH, can enhance SM biosynthesis. Salinity stress induced by NaCl in the nutrient solution enhanced the concentration of essential oils and phenolics in sweet basil (*Ocimum basilicum*) (Bernstein *et al.*, 2010; Corrado *et al.*, 2021b), and anti-malarial sesquiterpenoid artemisinin in *A. annua* (Qureshi *et al.*, 2005; Yadav *et al.*, 2017). Additionally, microbial elicitors can also be applied hydroponically to enhance SM biosynthesis (Aremu *et al.*, 2016; Partap *et al.*, 2020). Combining hydroponic elicitors, for instance microbial (*Aspergillus niger*), hormonal (methyl jasmonate), and silver nanoparticles, improved the bioactive and antioxidant activity in *Sylibium marianum* over individual application of these elicitors (Mubeen *et al.*, 2022). Hydroponic elicitation, therefore, can be an effective technique that can be implemented in CEP to improve MPs.

As with aerial factors, chronoculture can also be applied to the RZ through the timed application of treatments or diel regulation of factors such as nutrients or temperature (Eldridge *et al.*, 2020). However, the scope of RZ chronoculture for MPs remains to be fully explored. Finally, the type of soil-less cultivation itself can impact the levels of SMs in plants (Son *et al*., 2021). However, the causal elements affecting SMs based on the cultivation method remain to be determined.

Controlling the RZ environment to apply the proposed strategies offers a route to enhance SM biosynthesis in hydroponic CEP. However, manipulating the RZ is less straightforward than manipulating the aerial environment. Customizing nutrient levels in the nutrient solution requires real-time monitoring of nutrient ion concentrations, but is limited by technical constraints (Bamsey *et al.*, 2012). Moreover, responses to RZ manipulation can be species or cultivar specific (Zeljković *et al.*, 2022).

### Stacking the strategies

The proposed strategies can individually augment the concentration of target bioactive phytochemicals in MPs. However, their potential may be maximized when these strategies are ‘stacked’—implemented simultaneously in a cumulative or synergistic manner.

The strategic integration of environmental control strategies could amplify their benefits through their additive effects. For instance, consider combining the strategies of enhancing NPCA, elicitation, and chronoculture. The NPCA can first be increased through optimization of the environment. Subsequently, a stressor can be applied to plants with elevated NPCA to trigger the shunting of the additional assimilated carbon towards SM biosynthesis (Lavola *et al.*, 2000). Finally, the stressor can be applied at the optimal time of day, based on the circadian cycle, to further enhance the efficacy of elicitation. Another example of stacking is the enhancement of NPCA in conjunction with precise light control. This combination can significantly improve a plant’s capacity to efficiently utilize light for growth, while simultaneously signalling the production of appropriate SMs (Azad *et al.*, 2020; Karimi *et al.*, 2022). Therefore, it is reasonable to hypothesize that the additive effect of stacking multiple strategies can yield higher SM yields in MPs.

Stacking strategies facilitate the development of highly specialized environmental treatments that harness the responses of a given MP species to optimize resource use and enhance product quality. However, not all combinations are equally effective for all species, and multiple strategies could trigger complex pathways with potential trade-offs. Careful experimentation and monitoring can mitigate these trade-offs and identify effective strategy combinations for specific plant species and target metabolites.

## Challenges and outlook

**Fig. 6.**
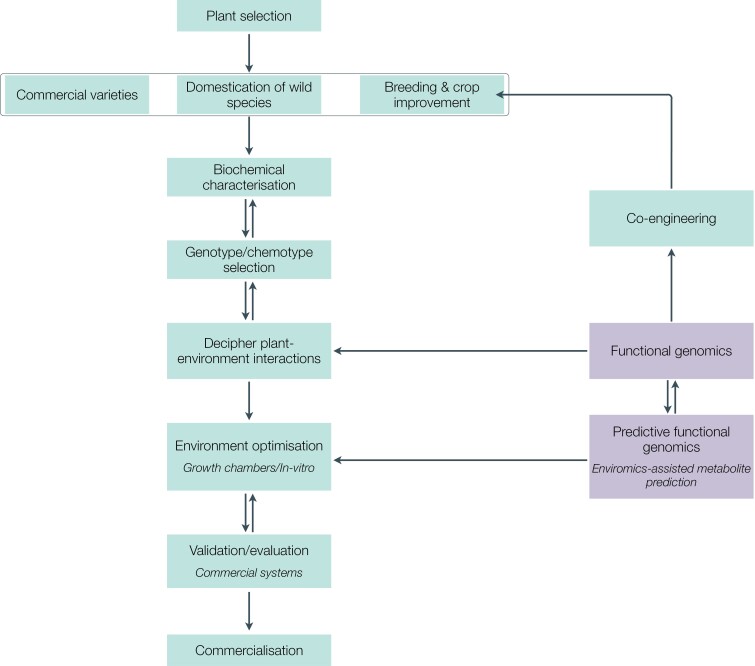
A proposed technical workflow for long-term implementation of controlled-environment phyto-pharmaculture. Medicinal plants are selected from commercially available genotypes, wild genotypes, or improved genotypes, and screened for their biochemical profiles using metabolomic techniques. The most desirable genotypes/chemotypes are selected for studies delineating the plant responses to environment treatments using functional genomic and multi-omic approaches. Co-engineering (Box 2) allows for simultaneous improvement in conditions of the growth environment and the genotypes of MPs. Based on the broader study findings, the environment conditions are optimized in growth chambers or *in vitro* cultures for a targeted metabolite profile with the assistance of predictive functional genomics. The optimized environment regime is validated in commercial systems for translation, persistence, and efficacy. The validated protocols with the selected genotype are adopted for large-scale commercial controlled-environment phyto-pharmaculture.

CEP holds the potential to enhance the production and quality of MPs by optimizing growth conditions. However, its widespread implementation faces challenges, necessitating further research that can aid the workflow for optimizing cultivation and improving the quality of MPs (Fig. 6). The primary challenge lies in the complexity of plant–environment interactions. Altering one environment variable can inadvertently affect others, leading to confounding effects on plant phytochemicals. For example, increasing light intensity can raise the canopy temperature. While high light intensity increases anthocyanin production in plants, the consequent increase in canopy temperature can offset anthocyanin production by reducing the activity of the enzyme phenylalanine ammonia-lyase (PAL) (Ayenew *et al.*, 2015). Certain environment treatments may also have synergistic effects, such as high temperature accelerating drought onset and enhancing flavonoid biosynthesis, as seen in Douglas fir (*Pseudotsuga menziesii*) (Jansen *et al.*, 2014). This complexity is particularly pronounced when considering multiple modifiable variables.

Given the complexities of plant metabolism, environmental interactions, species specificity, and treatment effects, fundamental research is needed to decipher the mechanisms underlying the proposed strategies (Fig. 6). It is crucial to understand how environmental treatments affect plant growth and SM yield, considering interactions and trade-offs (Canter *et al.*, 2005). Moreover, our understanding of the pathways regulating bioactive phytochemical production in MPs under various environmental conditions is limited. This gap can be addressed by integrating CEP with other technologies, such as functional genomics (Box 1).

Box 1. **Functional genomics for controlled-environment phyto-pharmaculture (CEP)**

Functional genomics, in conjunction with CEP, offers an effective approach for deciphering the complex metabolic responses of plants to various environmental control strategies. A range of functional genomic techniques, including transcriptomics, proteomics, metabolomics, and enviromics, can help establish links between the activity of specific genes, molecular signatures, and the desired biochemical profile under a specific environmental condition (Weston *et al.*, 2008; Marondedze *et al.*, 2018). These techniques can also shed light on the coordinated regulation of biosynthetic pathways in response to different environmental treatments or elicitors (Yamashita *et al.*, 2023).

Functional genomics also encompasses enviromics, which involves measuring and analysing environment conditions relevant to plant growth and metabolism. Controlled-environment systems, which continually monitor the environment, generate invaluable environment data that can be used for plant improvement. By integrating the entirety of the measured environment data—the envirome—with plant genomic, transcriptomic, metabolomic, and phenomic responses, researchers can investigate the effects of specific environmental factors on the phytochemical profiles at various levels (Costa-Neto and Fritsche-Neto, 2021). We therefore envisage the technique of enviromics-aided metabolomic prediction, which integrates enviromic and metabolomic or transcriptomic data to predict the plant metabolite responses to specific environment conditions.

The integration of functional genomics into CEP enables precise tailoring of environmental regimes for desired biochemical profiles in MPs. Therefore, the application of functional genomics to CEP can offer a comprehensive understanding of how various environmental conditions and strategies influence plant growth and medicinal value, ultimately optimizing cultivation protocols and enhancing the quality of MPs.

Another significant challenge is that most wild and cultivated MPs are not well suited for indoor cultivation due to their slow growth rate, low germination, dependence on symbiotic microbes, and limited adaptability to stacked hydroponic systems. Moreover, specific phenotypes lend themselves well to indoor cultivation in controlled environments. These phenotypes include, but are not limited to, dwarf stature, root structure suited for hydroponics, fast growth cycle, enhanced resource (light, nutrients, water, and CO_2_) use efficiency, tolerance to low light, fruits amenable to automated harvesting, and self-pollination (Folta, 2019; O’Sullivan *et al.*, 2020; Kwon, 2023). While the phenotypes of wild plants can be modified modestly through environmental modulation (Carvalho and Folta, 2014; SharathKumar *et al.*, 2020), realizing the full potential of MPs, most of which are non-domesticated, requires targeted breeding and genetic improvements to introduce traits essential for indoor cultivation (Fig. 6). This also involves breeding or selecting plant genotypes that are more amenable to environment modification, exhibiting a wide range of desired phenotypes and biochemical responses (Carvalho and Folta, 2014).

Besides targeting phenotypes desirable for indoor cultivation, breeders must also improve the medicinal value, stability, uniformity, and yield of bioactive compounds. The -omic data associated with desirable phenotypes derived through functional genomics (as discussed in Box 1) can be applied to improve crops using molecular and genome editing techniques (Scheben *et al.*, 2017; Varshney *et al.*, 2021). Furthermore, incorporating molecular breeding, metabolic pathway engineering, and *in vitro* techniques can aid in the domestication of MPs (Canter *et al.*, 2005). Notably, CEP offers an opportunity to expedite the breeding and crop improvement of MPs through co-engineering, which involves simultaneous and complementary optimization of both the growth environment and plant genetics (Box 2).

Box 2. **Co-engineering plants and the environment using controlled environments**

Co-engineering refers to the simultaneous optimization of plant genetics and growth environment. This approach allows for fine-tuning of environmental conditions to complement specific genetic modifications. For instance, a plant genetically engineered for enhanced photosynthesis can thrive in an environment with precisely controlled light spectra and intensity. By carefully designing the growing environment to match the specific needs of a particular plant species and modifying the plant genetics to better adapt to controlled environments, it is possible to accelerate the improvement in growth rates, yield, and standardized medicinal quality of plants. This approach of coordinated engineering of the environment along with plant genetics can enhance the synergy, precision, and pace of crop improvement and domestication for CEP.

Co-engineering will fundamentally involve modelling and predicting the interactions between the genotype and environment to tailor the combination. Omics-based modelling in controlled environments can help elucidate the genotype×environment interactions under specific environmental treatments to identify the ideal combination for a desired phenotype. A recent study by Yamashita *et al.* (2023) demonstrates this approach by modelling the genotype×environment interactions between 14 lettuce genotypes and four light spectra on the morphological, biochemical, and transcriptomic responses. The modelled genotype×environment interaction was then used to co-optimize the genotype and environment to maximize the lettuce crop performance in controlled environments. This approach can be applied not only to select ideal MP genotypes with desirable morphological and biochemical responses for CEP, but also to identify desirable genes for targeted breeding.

Functionally, the successful application and stacking of environmental control strategies in large-scale commercial CEP can be an intractable challenge. Non-destructive imaging technologies (e.g. fluorescence, RGB, and hyperspectral imaging platforms) combined with machine learning (ML) algorithms can help overcome this challenge. For instance, image-based phenotyping enables the estimation of coloured metabolites, such as anthocyanins and carotenoids in plant leaves (Gitelson *et al.*, 2006). Automated monitoring enables real-time data collection based on plant and environmental conditions, enhancing precision in chronocultural operations. Phenotyping can also be applied to plant roots, as demonstrated by the workflow developed by Chen *et al.* (2023) that estimates tanshinone contents based on root morphology in *S. miltiorrhiza*. Additionally, imaging technologies can also measure growth rate, stress levels, and photosynthetic performance in plants (Humplík *et al.*, 2015; Singh *et al.*, 2021). Data from these platforms can be used to fine-tune the environmental control strategies applied for enhancing the yield of target compounds. Furthermore, imaging and ML can also be integrated with functional genomics and co-engineering in deciphering the genotype×environment interaction to improve MPs for CEP (Ghanem *et al.*, 2015). Therefore, image-based phenotyping would be an essential tool towards realizing large-scale CEP. However, this nascent technology needs further development and validation for CEP.

Lastly, several ancillary yet critical questions need to be answered. Is CEP environmentally and economically feasible? Do the MPs produced in CEP truly surpass those of the conventionally produced plants in terms of their pharmacological efficacy? What are the effective regulatory frameworks and cultivation guidelines for CEP? How would the public perceive the commercial-scale controlled-environment production of MPs that are associated with ethnocultural identities? As CEP assumes a pivotal role in commercial MP production, addressing these questions should complement biological advancements.

## Conclusion

By leveraging controlled environments and proposed strategies, we envisage a sustainable approach to meet the growing global demand for safe, high-quality MPs with consistent metabolite profiles. The proposed CEP strategies essentially come down to regulating plant physiology and metabolism. Understanding how various environmental cues regulate metabolite flux, SM biosynthesis, and gene actions can have a far-reaching impact in the fields of metabolism engineering, ecology, and human nutrition, besides plant medicine. Thus, we emphasize achieving a comprehensive understanding of plant–environment interactions and the underlying mechanisms that govern the phytochemical responses of MPs. Plant biological research will, therefore, play a pivotal role towards the effective application of CEP, delivering benefits for the pharmaceutical and healthcare sectors, as well as for individuals who rely on MPs for their health and well-being.
